# QoL analyses from INFORM study, a phase III study of gefitinib versus placebo as maintenance therapy in advanced NSCLC

**DOI:** 10.1038/srep11934

**Published:** 2015-07-03

**Authors:** Yun-Peng Yang, Yu-Xiang Ma, Yan Huang, Yuan-Yuan Zhao, Wen-Feng Fang, Shao-Dong Hong, Ying Tian, Cong Xue, Jin Sheng, Li Zhang

**Affiliations:** 1 State Key Laboratory of Oncology in South China, Collaborative Innovation Center for Cancer Medicine, Sun Yat-sen University Cancer Center, Guangzhou, Guangdong, China

## Abstract

This report aimed to provide the full results of QoL assessment in INFORM study. QoL was assessed by FACT-L questionnaire. QoL improvement ratio in gefitinib arm was higher than placebo arm (FACT-L: 46% vs. 22%, *p* < 0.001; TOI: 41% vs. 18%, *p* < 0.001; LCS: 46% vs. 22%, *p* < 0.001). Gefitinib prolonged time-to-worsening of QoL (FACT-L: 2.8 m vs 1.4 m, *p* = 0.019; TOI: 3.5 m vs 1.4 m, *p* = 0.006; LCS: 2.8 vs 1.4 m, *p* = 0.028). Patients with an improvement in QoL had longer PFS (FACT-L: 9.4 m vs. 2.8 m vs. 2.7 m, *P* < 0.001; TOI: 9.9 m vs. 2.8 m vs. 2.1 m, *P* < 0.001; LCS: 9.4 m vs. 2.9 m vs. 2.1 m, *P* < 0.001) and OS (FACT-L: 25.4 m vs. 19.9 m vs. 14.4 m, *P* = 0.003; TOI: 25.7 m vs. 19.0 m vs. 12.7 m, *P* = 0.002; LCS: 25.4 m vs. 19.3 m vs. 14.7 m, *P* = 0.004) compared with patients with stable or worsened QoL. Furthermore, in patients with good QoL at baseline, the treatment of gefitinib couldn’t improve OS compared to placebo, whereas patients with low QoL experienced marginal significant improvement in OS (20.6 m vs 14.4, *p* = 0.051). Our study indicated that gefitinib could improve patients’ QoL, confirmed the prognostic value of QoL changes during treatment, and implied patients with low QoL at baseline may be the potential population which will gain OS benefit from maintenance EGFR-TKI therapy.

Lung cancer continues to remain the most frequently diagnosed cancer and the leading cause of cancer death worldwide^1^. Non-small-cell lung cancer (NSCLC) is the most common form of lung cancer, accounting for ∼85% of all cases, with five-year survival rate <20%^2^. Although platinum-doublet chemotherapy has demonstrated to prolong overall survival, the prognosis remains to be poor^3^,^4^. Recent progress in targeted therapy has provided new therapy options to treat NSCLC. The epidermal growth factor receptor (EGFR) pathway is one of the most important signaling pathways in NSCLC^5^,^6^. Previous studies have proven the remarkable effectiveness of EGFR tyrosine kinase inhibitors (TKIs) such as gefitinib (Iressa, AstraZeneca, Macclesfield, United Kingdom) in treatment of NSCLC^7^,^8^,^9^,^10^.

Maintenance therapy with the goal of improving progression-free survival and overall survival is given to patients who have achieved a sufficient response or stable disease (SD) during induction therapy. According to the results of several studies, patients with locally advanced or metastatic (stage IIIB to IV) NSCLC who receive maintenance therapy have a better overall prognosis than those who don’t receive maintenance therapy^11^,^12^,^13^,^14^,^15^,^16^. The INFORM study (registered with Clinicaltrials.gov on October 9, 2008, number NCT00770588) was a double-blind, randomised, parallel-group study comparing the efficacy and safety of gefitinib (250 mg/day) with placebo as maintenance therapy in Chinese patients with locally advanced or metastatic NSCLC. The efficacy and safety results of this study have been reported previously^17^. Progression-free survival (PFS) was significantly longer with gefitinib than with placebo (4.8 months vs 2.6 months; hazard ratio [HR] 0.42, 95% CI 0.33–0.5; p < 0.0001). Overall survival (OS) was similar between the two arms.

Patients with NSCLC often suffer from a variety of distressing symptoms, which are usually present at diagnosis and last throughout the course of the disease, impairing patients’ health-related quality of life (HRQoL) profoundly^18^,^19^,^20^. Therefore improvement of HRQoL and symptoms is particularly important when treating patients with NSCLC. HRQoL evaluation was a preplanned secondary objective of the INFORM study. Here, we present the full results of the HRQoL assessment in INFORM. Additionally, the relationship between the change in health-related quality of life score and prognosis was analyzed. Furthermore, we examined the treatment effect of gefitinib versus placebo on PFS and OS by baseline HRQoL.

## Results

### Baseline patient characteristics and QoL assessments

**Figure 1:**
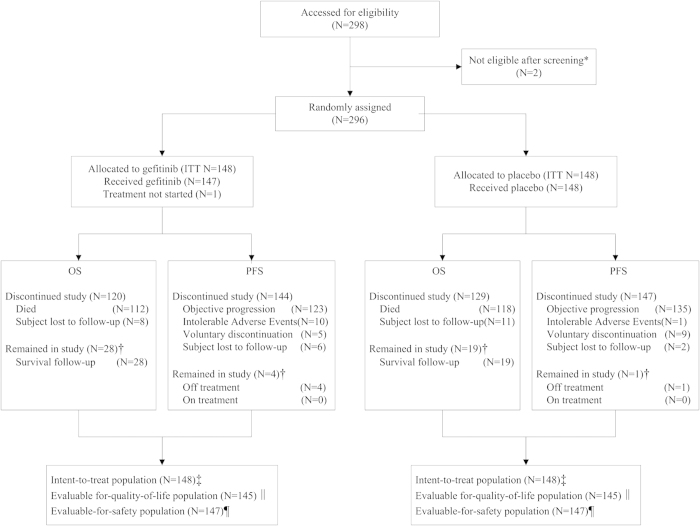
CONSORT diagram. OS = overall survival, PFS = progression free survival, ITT = intend-to-treat. (*)Two patients discontinued the study before randomization. (†) Cut off dates: June 17, 2014, for overall survival (OS) and progression-free survival (PFS). (‡) All patients who were randomly assigned to a study group were included in the intent-to-treat (ITT) analysis. (¶) All patients who received at least one dose of study treatment were included in the safety analysis. (‖)All patients received quality of life assessment at baseline were included in QoL analysis.

Of the 296 patients randomized, the evaluable for quality-of-life (EFQ) analyses population is 290 (gefitinib arm 145, placebo arm 145). The EGFR-mutation status is known in 79 patients (30 positive: gefitinib arm 15, placebo arm 15; 49 negative: gefitinib arm 25, placebo arm 24). Patient disposition is presented in Fig. 1. The key demographics and characteristics of the ITT population and the EFQ population are shown in Table 1. The percentages of basic characteristics like age, gender, histology type, smoking status, disease stage, PS, smoking history and those who received treatment with 1st line chemotherapy are comparable between EFQ and ITT population, and also show a favorable equilibrium in gefitinib and placebo group.

**Figure 2:**
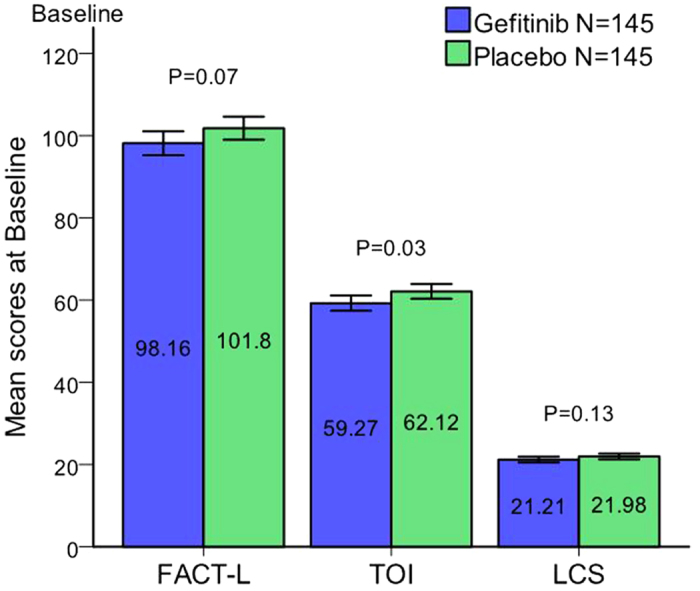
Bar charts of the quality of life status of each arm at baseline. 290 patients were evaluable for quality-of-life (EFQ) analyses at baseline (gefitinib arm 145, placebo arm 145). FACT-L and LCS scores are comparable between the two arms, while the TOI score in placebo arm is significantly higher than that in gefitinib arm.

The results of QoL assessment at baseline of each arm are shown in Fig. 2. FACT-L and LCS scores are comparable between the two arms, while the TOI score in placebo arm is significantly higher than that in gefitinib arm.

### Change of QoL from baseline during treatment

**Figure 3:**
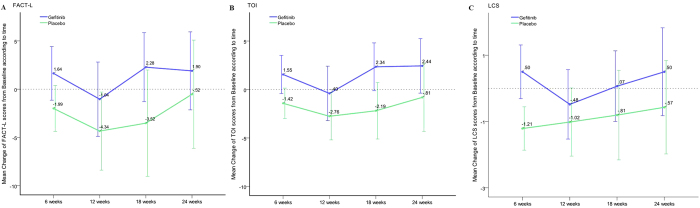
Mean change of quality of life (QoL) from baseline to 24 weeks in gefitinib and placebo arms (**A**: FACT-L scores; **B**: TOI scores; **C**: LCS scores). The QoL changes from baseline were calculated by each arm every 6 weeks until less than 10% of patients had available data. The gefitinib arm always performed better than the placebo arm in FACT-L, TOI and LCS during the cause of treatment.

Considering the change of QoL from baseline at every visit, the gefitinib arm always performed better than the placebo arm in FACT-L, TOI and LCS. (Fig. 3)

**Figure 4:**
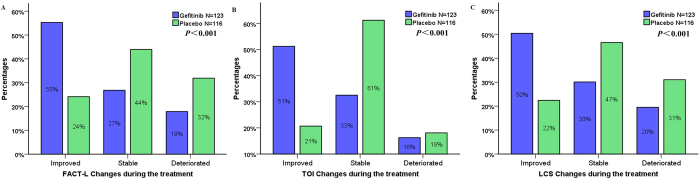
The proportion of patients with different quality of life (QoL) change during the treatment accodring to the results of FACT-L (**A**), TOI (**B**) and LCS (**C**). The change in QoL score were analyzed for patients with a baseline and at least one post-baseline QoL assessment (123 in gefitinib arm and 116 in placebo arm). The improvement ratios in gefitinib arm were significantly higher than placebo arm.

As mentioned in the methods section, we defined the patients’ QoL change during the treatment as improved, stable or deteriorated. As illustrated by Fig. 4, the improvement ratios in gefitinib arm are significantly higher than placebo arm (FACT-L: 55% vs. 24%, *p* < 0.001; TOI: 51% vs. 21%, *p* < 0.001; LCS: 50% vs. 22%, *p* < 0.001). Additionally, gefitinib also prolonged time-to-worsening of QoL when compared with placebo (FACT-L: 2.8 m vs 1.4 m, *p* = 0.019; TOI: 3.5 m vs 1.4 m, *p* = 0.006; LCS: 2.8 vs 1.4 m, *p* = 0.028).

Additionally, in patients positive for EGFR mutation (15 in gefitinib arm and 15 in placebo arm), gefitinib tended to increase the ratio of QoL improvement (TOI: 60% vs. 20%, *p* = 0.060), and prolong time-to-worsening of QoL (TOI: 7.3 m vs 1.9 m, *p* = 0.055) when compared with placebo. The difference was not statistically significant might due to the small number of the patients. However, in patients with wild-type EGFR gene (25 in gefitinib arm and 24 in placebo arm), the ratio of QoL improvement (TOI: 32% vs. 25%, *p* = 0.588) and time-to-worsening of QoL (TOI: 2.1 m vs 1.5 m, *p* = 0.380) were comparable during treatment between the two arms.

### Relationship between the change in QoL and prognosis

**Figure 5:**
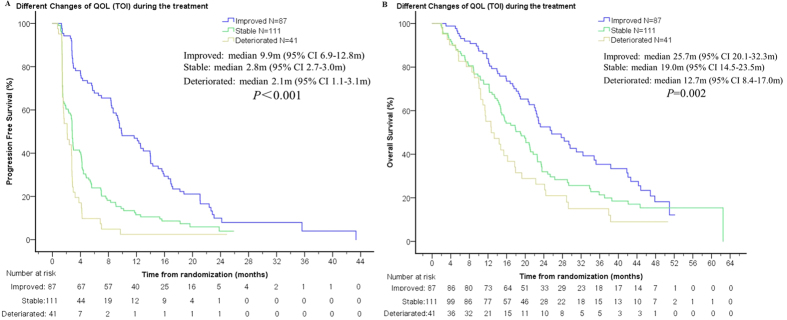
The relationship between QoL change (assessed by TOI score) during treatment and prognosis (**A**: PFS; **B**: OS). Patients with an improvement in QoL had significantly longer PFS and OS compared with patients with stable or worsened QoL.

The relationship between the change in QoL score and prognosis was analyzed according to subgroups with different QoL alterations. We found that patients with an improvement in QoL had significantly longer PFS (FACT-L: 9.4 m vs. 2.8 m vs. 2.7 m, *P* < 0.001; TOI: 9.9 m vs. 2.8 m vs. 2.1 m, *P* < 0.001; LCS: 9.4 m vs. 2.9 m vs. 2.1 m, *P* < 0.001) and OS (FACT-L: 25.4 m vs. 19.9 m vs. 14.4 m, *P* = 0.003; TOI: 25.7 m vs. 19.0 m vs. 12.7 m, *P* = 0.002; LCS: 25.4 m vs. 19.3 m vs. 14.7 m, *P* = 0.004) compared with patients with stable or worsened QoL. Figure. 5 illustrated the relationship between TOI change and prognosis.

### Treatment effect of gefitinib versus placebo on PFS and OS by baseline QoL

**Figure 6:**
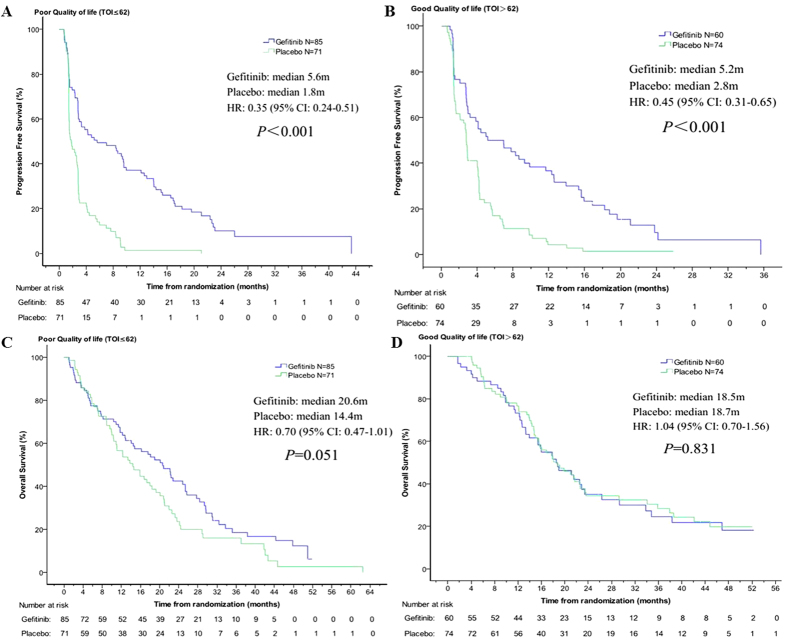
Kaplan-Meier curves for progression-free survival in patients with poor QoL (**A**) and good QoL (**B**), and for overall survival in patients with poor QoL (**C**) and good QoL (**D**). Patients had been divided into good or poor QoL group according to the score of TOI at baseline (good: TOI > 62; poor: TOI ≤ 62). Cox proportional hazards regression model were used to estimate hazard ratio (HR). HR < 1 implied a lower risk of progression or death with gefitinib than with placebo.

In our study, patients had been divided into good or poor QoL group according to the score of TOI at baseline. The key demographic and characteristics of each group was summarized in Table 2. Then the treatment effect of gefitinib versus placebo on prognosis by baseline QoL was analyzed. As shown in Fig. 6, the results indicate that PFS might not be affected by baseline QOL status, and the gefitinib arm shows a significant longer PFS in both poor QoL and good QoL status than placebo arm. However, the benefit in OS from gefitinib treatment was affected by baseline QoL status. In patients with good QoL at baseline, the treatment of gefitinib could not improve OS compared to placebo (18.5 vs. 18.7 months, *P* = 0.831), whereas patients with low QoL experienced marginal significant improvement in OS (20.6 m vs 14.4, *p* = 0.051).

## Discussion

In the INFORM study, although progression-free survival was significantly longer with gefitinib than with placebo, overall survival was similar between the two arms. However, according to our study, gefitinib as maintenance when compared with placebo conferred statistically significant improvements in FACT-L, TOI, LCS, proportion of patients with clinical relevant improvement, and time to worsening of QoL. Considering that preserving or improving the patients’ QOL is another important goal of treating advanced NSCLC in addition to clinical benefits such as longer progression free survival, higher tumor response rates, and prolonged overall survival, the results of our study could provide some useful information for clinical practice.

It’s worth noting that there was a clear decrease in total FACT-L, TOI, and LCS scores of gefitinib arm at 12 weeks. Previous studies demonstrated that the benefit of gefitinib was limited to patients with EGFR mutations^7^,^10^. INFORM study also found that the PFS was similar between gefitinib and placebo arms for patients with EGFR mutation-negative tumors (2.7 m vs 1.5 m, HR 0.86, 95% CI 0.48-1.51)^17^. Therefore, about half of the patients with EGFR mutation-negative tumors on gefitinib would have disease progression and experience QoL and symptoms worsening at 12 weeks, thus adversely affect the QoL and symptom scores of the whole gefitinib arm.

The INFORM study did not require molecular selection for patient’s enrolment due to lack of evidence to support the predictive value of EGFR mutation status at the initiation of the study. Therefore, the EGFR mutation status was detected in only 79 patients of the study. However, it’s well known that the EGFR mutation rate in East-Asian population is much higher than that in Western population, and 30–40% of the patients from East-Asian would harbor EGFR mutations^21^,^22^,^23^. Several studies have confirmed that EGFR-TKIs could significantly improve the QoL of patients with EGFR mutation-positive tumors^24^,^25^. Our study also implies that in patients positive for EGFR mutation, gefitinib tends to improve the QoL, increase the ratio of QoL improvement, and prolong time-to-worsening of QoL. Therefore, patients with EGFR mutations could benefit from gefitinib treatment, and their improvement in QoL might lead to the positive change of the whole gefitinib arm in our study.

The relationship between changes in QoL scores from baseline during chemotherapy and prognosis has been analyzed in a few studies, which has found that changes in QoL could predict survival in NSCLC patients^26^,^27^,^28^. Our study confirmed the prognostic value of changes in QoL scores during EGFR-TKI treatment for both PFS and OS. Patients whose QoL was improved during treatment had statistically significant longer PFS and OS than patients whose QoL was stable or deteriorated. These findings of our work suggest that regular QoL assessments could be necessary during the course of EGFR-TKI treatment to provide valuable information about the prognosis of the patients. When a patient’s QoL begins to deteriorate, which may indicate disease progression and poor overall survival, appropriate intervention should be considered.

Since there has been a lack of clinical trials conducted to answer the question as to which patients will gain the greatest benefit from maintenance therapy versus delayed second-line treatment, there has been debate regarding appropriate candidates for immediate maintenance therapy. Some physicians believe that patients with low QoL and high symptom burden should be considered to receive maintenance therapy, because they are at risk of rapid disease progression and symptom deterioration, which could impair their ability to receive additional lines of treatment^29^,^30^,^31^. On the other hand, patients with better QoL and few symptoms could wait to receive second-line therapy after disease progression. However, according to the analysis from JMEN study, significantly longer OS for pemetrexed maintenance therapy versus placebo occurred only in low symptom burden patients and PS 0 patients rather than high symptom burden or PS 1 patients^32^. Additionally, exploratory subgroup analysis of IFCT-GFPC 0502 study also showed that OS benefit from maintenance gemcitabine treatment might only concern patients with a PS of 0 after induction chemotherapy^33^. Thus patients with better Qol and performance status seem to be the target population of maintenance therapy. Surprisingly, our study had a different result. We found that patients with worse QoL (TO ≤ 62) experienced a statistically significant improvement in PFS (5.6 m vs 1.8 m, *p* < 0.001) and marginal significant improvement in OS (20.6 m vs 14.4, *p* = 0.051), whereas patients with better QoL (TOI > 62) experienced significant improvement only in PFS (9.8 m vs 2.8 m, *p* < 0.001) but not in OS (18.5 m vs 18.7 m, *p* = 0.831) when treated with gefitinib versus placebo. The incontinence between the results of our study and the previous studies might be explained by the difference of toxicities between chemotherapy and EGFR-TKI. Considering that chemotherapy has a worse tolerability than EGFR-TKI, requiring better performance status and QoL to tolerate the toxicities, thus patients with poor performance status and high symptom burden at baseline may not benefit from maintenance chemotherapy. However, these patients might derive benefit from maintenance EGFR-TKI due to its favorable toxicities profile.

Our study has several limitations. First, the number of patients with known EGFR mutation status (79/296, 26.7%) is insufficient for subgroup analyses according to EGFR mutation. Additionally, as a post hoc study, the QoL analyses are not statistically powered. Thus prespecified and appropriately powered analyses are warranted in the future to validate the findings of our study.

In conclusion, the results of our study indicate that gefitinib as maintenance could significant improve patients’ QoL when compared with placebo, confirm the prognostic value of changes in QoL scores during EGFR-TKI treatment, and imply patients with low QoL at baseline may be the potential population which will gain OS benefit from maintenance EGFR-TKI therapy.

## Methods

### Study design

Full details of the INFORM study design (NCT00770588) have been published previously^17^. Eligible patients were 18 years or older and had a life expectancy of more than 12 weeks, histologically or cytologically confirmed stage IIIb or IV NSCLC, a WHO performance status of 0–2, and completed four cycles of first-line platinum-based doublet chemotherapy without disease progression and unacceptable toxicities.

Eligible patients were randomized 1:1 to gefitinib (250 mg/day orally) or placebo (orally) administered 3–6 weeks post-chemotherapy. Treatment continued until objective disease progression, intolerable toxicity, dose delay/interruption for >14 days, withdrawal of consent, or serious non-compliance with study protocol.

All patients provided written, informed consent, with separate consent obtained for optional provision of tumor material for biomarker analyses. The study was conducted in accordance with the Declaration of Helsinki, the International Conference on Harmonization/Good Clinical Practice, applicable regulatory requirements, and AstraZeneca’s policy on bioethics. The approval of this study on patients’ QoL was obtained from independent ethics committee of Sun Yat-Sen University Cancer Center.

The primary endpoint of INFORM was superiority of gefitinib relative to placebo in terms of progression free survival (PFS). Overall survival (OS) and QoL analyses were included in secondary endpoints.

### Quality of life assessment

Patients received QoL assessment after randomization before first drug dose, and then at each visit (every 6 weeks) during progression free survival. QoL were evaluated by the Functional Assessment of Cancer Therapy-Lung (FACT-L) questionnaire^34^. The FACT-L questionnaire contains 34 items which rated on a 5-point Likert scale, includes four dimensions (physical well-being, PWB; social/family well-being, SWB; emotional well-being, EWB and functional well-being, FWB) and Lung Cancer Subscale (LCS). FACT-L total score (the sum of all five domains), Trial Outcome Index (TOI; the sum of the physical, functional well-being, and LCS domains), and LCS were all used to assess the change of QoL in our study.

### Statistical analyses

All patients with evaluable QoL assessment at baseline were considered as evaluable-for-quality-of-life (EFQ) population. The change in QoL score would be analyzed for patients with a baseline and at least one post-baseline QoL assessment. Different distribution of patients in every basic characteristics category was test by Pearson’s chi-square for balancing. The difference of baseline QoL score between the two groups was tested by two-sample *t*-test. The changes from baseline for FACT-L total score, TOI, and LCS were calculated by randomized treatment group, for every 6weeks that QoL was assessed where 10% or more patients had available data. In our study, the best overall response of QoL during the treatment was calculated for FACT-L, TOI, and LCS scores. A clinically relevant improvement was defined as an increase from baseline of 6 or more points for FACT-L and TOI, and 2 or more points for LCS. Clinically relevant deterioration was defined as a decrease from baseline of 6 or more points for FACT-L and TOI, and 2 or more points for LCS. Otherwise would be defined as stable. The improvement, stable or deterioration rate was calculated for each treatment group as a percentage of the total number of patients with improved, stable, or deteriorated QoL during treatment, respectively. Improvement rates were compared between treatment groups using Pearson’s chi-square test. In the present study, time-to-worsening of FACT-L, TOI, and LCS was defined as the interval from randomization to the first visit of “worsened”, and was presented by different treatment groups with median values and 95% CIs and by Kaplan-Meier plots. PFS and OS were analyzed by using the unadjusted Cox proportional hazards regression model to estimate hazard ratios (HRs) and 95% CIs. Kaplan-Meier curves were used to estimate survival. Differences in survival estimates between subgroups were assessed by using log-rank test. All significance levels refer to two-sided tests. A *p* value of <0.05 was considered significant.

## Additional Information

**How to cite this article**: Yang, Y.-P. *et al*. QoL analyses from INFORM study, a phase III study of gefitinib versus placebo as maintenance therapy in advanced NSCLC. *Sci. Rep*. **5**, 11934; doi: 10.1038/srep11934 (2015).

## Figures and Tables

**Table 1:** Key Demographic and Baseline Characteristics (EFQ and ITT Populations).

Category	ITT Population (N = 296)		EFQ population (N = 290)	
	Gefitinib % (N = 148)	Placebo % (N = 148)	Gefitinib % (N = 145)	Placebo % (N = 145)
Median age (Range)	55 (31–79)	55 (20–75)	54 (31–79)	54 (20–75)
Gender				
Male	83 (56)	92 (62)	81 (56)	90 (62)
Female	65 (44)	56 (38)	64 (44)	55 (38)
Histology type				
Adenocarcinoma	105 (71)	104 (70)	104 (72)	104 (72)
Squamous	27 (18)	30 (20)	25 (17)	27 (19)
Others	16 (11)	14 (10)	16 (11)	14 (10)
Disease stage				
IIIB	42 (29)	32 (30)	40 (28)	30 (21)
IV	106 (71)	116 (70)	105 (72)	115 (79)
WHO PS				
0	69 (47)	72 (49)	68 (47)	70 (48)
1	76 (51)	72 (49)	74 (51)	71 (49)
2	3 (2)	4 (3)	3 (2)	4 (3)
Smoking history				
Non smoker	79 (53)	81 (55)	78 (54)	80 (55)
Ex-smoker	57 (39)	55 (37)	56 (39)	54 (37)
Current smoker	12 (8)	12 (8)	11 (8)	11 (8)
Type of 1^st^ Chemotherapy				
Taxane †	60 (41)	66 (45)	59 (41)	65 (45)
Non-taxane ‡	88 (59)	82 (55)	86 (59)	80 (55)
Response to 1^st^ Chemotherapy				
PR or CR	58 (39)	51 (34)	56 (39)	49 (34)
SD	90 (61)	97 (66)	89 (61)	96 (66)
EGFR Mutation Status				
Positive	15 (10)	15 (10)	15 (10)	15 (10)
Negative	25 (17)	24 (16)	25 (17)	23 (16)
Unknown	108 (73)	109 (74)	105 (72)	107 (74)

Data are years (range) or number (%) as appropriate. ITT = intention-to-treat, EFQ = enable-for-quality-of-life, PS = performance status, PR = partial response, CR = complete response, SD = stable disease, EGFR = epidermal growth factor receptor.

^†^Includes docetaxel and paclitaxel.

^‡^Includes gemcitabine, vinorelbine, and navelbine.

**Table 2:** Key Demographic and Baseline Characteristics in Good and Poor QOL status (EFQ Populations).

Category	Good Quality of life at baseline (FACT-TOI > 62) N = 134*			Poor Quality of life at baseline (FACT-TOI ≤ 62) N = 156*		
	Gefitinib % (N = 60)	Placebo % (N = 74)	†*P* value	Gefitinib % (N = 85)	Placebo % (N = 71)	†*P* value
Median age (Range)	56 (32–74)	53 (20–72)	*0.115*	53 (31–79)	55 (33–75)	*0.359*
Gender						
Male	40 (67)	47 (64)		41 (48)	43 (61)	
Female	20 (33)	27 (36)	*0.720*	44 (52)	28 (39)	*0.148*
Histology type						
Adenocarcinoma	42 (70)	56 (76)		63 (85)	51 (72)	
Squamous	11 (18)	13 (18)	*0.592*	14 (16)	14 (20)	*0.863*
Others	7 (12)	5 (6)		8 (9)	6 (8)	
Disease stage						
IIIB	19 (32)	11 (15)		21 (25)	19 (27)	
IV	41 (68)	63 (85)	*0.023*	64 (75)	52 (73)	*0.854*
WHO PS						
0	33 (55)	40 (54)		35 (41)	30 (42)	
1	26 (43)	34 (46)	*0.525*	48 (56)	37 (52)	*0.541*
2	1 (2)	0 (0)		2 (3)	4 (6)	
Smoking history						
Non smoker	28 (47)	40 (54)		50 (59)	40 (56)	
Current			*0.487*			*0.871*
or Ex-smoker	32 (53)	34 (46)		35 (41)	31 (44)	
Type of 1^st^ Chemotherapy						
Taxane	28 (47)	30 (41)		31 (36)	35 (49)	
Non-taxane	32 (53)	44 (59)	*0.489*	54 (64)	36 (51)	*0.143*
Response to 1^st^ Chemotherapy						
PR or CR	27 (45)	31 (42)		29 (34)	18 (25)	
SD	33 (55)	43 (58)	*0.729*	56 (66)	53 (75)	*0.294*
EGFR Mutation Status						
Positive	8 (13)	7 (9)		7 (8)	8 (11)	
Negative	7 (12)	9 (12)	*0.779*	18 (21)	14 (20)	*0.809*
Unknown	45 (75)	58 (78)		60 (71)	49 (69)	

Data are years (range) or number (%) as appropriate. EFQ = enable-for-quality-of-life.

^*^Good QOL and poor QOL: TOI score is the basis of QOL division, and overall survival conducted as the outcome indicator. The receiver operating characteristic (ROC) curve was used to find the cut-off point of TOI score, which have the highest sensitivity and specificity to indicate OS.

^†^Pearson’s chi-square test was used to analyze between the two groups, Fisher’s exact test if n. ≤ 5.QOL = quality of life, FACT-TOI = functional assessment of cancer therapy - trial outcome index.
